# Belonging and Health Outcomes in Older Americans: A Scoping Review

**DOI:** 10.1093/geroni/igaf122.3721

**Published:** 2025-12-31

**Authors:** Charlotte Moss, Zander Vasquez, Raj Shah

**Affiliations:** Rush University Medical Center, Chicago, Illinois, United States; Rush Medical College at Rush University, Chicago, Illinois, United States; Rush University Medical Center, Chicago, Illinois, United States

## Abstract

As the number of older Americans grows, there is a movement to reframe aging as being about extending healthspan rather than just life expectancy. A sense of belonging has been theorized to be a positive influence on healthy longevity. This scoping review examined how belonging is associated with mental and physical health outcomes in older adults. We conducted a literature search on PubMed/MEDLINE, Scopus, PsycINFO, CENTRAL, and Google Scholar for studies published between 2015 and 2025. We followed the PRISMA Scoping Review guidelines and registered our protocol. We included original research articles conducted in the United States conceptualizing belonging as a predictor of various health behaviors or outcomes. We excluded studies not available in English, lacking a measure of belonging, and whose populations included individuals under 50 years old. After 1167 titles and abstracts and 88 full manuscripts were triaged, our search yielded 23 studies. Almost half of the articles examined thwarted belongingness and found it was a significant predictor of suicidality. Other outcomes included depression, loneliness, life satisfaction, and physical health. Belonging—or its absence—significantly predicted mental health outcomes, though its effect on physical health were mixed. These findings highlight associations of belonging and health outcomes in older Americans. Few articles studied the direct effects of belonging on chronic health conditions and longevity, highlighting gaps in the literature. More research on this topic is warranted to delineate if sense of belonging is a modifiable resilience factor for increasing healthspan.

